# The Inhibitory Effects of New Zealand Pine Bark (Enzogenol^®^) on α-Amylase, α-Glucosidase, and Dipeptidyl Peptidase-4 (DPP-4) Enzymes

**DOI:** 10.3390/nu14081596

**Published:** 2022-04-12

**Authors:** Wen Xin Janice Lim, Cheryl S. Gammon, Pamela von Hurst, Lynne Chepulis, Rachel A. Page

**Affiliations:** 1 School of Health Sciences, Massey University, Auckland 0632, New Zealand; w.x.j.lim@massey.ac.nz (W.X.J.L.); c.gammon@massey.ac.nz (C.S.G.); 2 Riddet Institute, Massey University, Palmerston North 4442, New Zealand; 3 School of Sport, Exercise and Nutrition, Massey University, Auckland 0632, New Zealand; p.r.vonhurst@massey.ac.nz; 4 Waikato Medical Research Centre, Te Huataki Waiora School of Health, University of Waikato, Hamilton 3216, New Zealand; lynne.chepulis@waikato.ac.nz; 5 School of Health Sciences, Massey University, Wellington 6021, New Zealand; 6 Centre for Metabolic Health Research, Massey University, Auckland 0632, New Zealand

**Keywords:** functional food, hyperglycaemia, hypoglycaemic effects, impaired glycaemic control, incretin effect, polyphenol, starch inhibition

## Abstract

The New Zealand pine bark extract (Enzogenol^®^) has previously been shown to elicit acute hypoglycaemic effects in humans. The present study investigated the underlying mechanisms of Enzogenol^®^ in reducing postprandial glucose in humans. The potential inhibitory action of Enzogenol^®^ against digestive enzymes: α-amylase and α-glucosidase, and dipeptidyl peptidase-4 (DPP-4) enzyme was determined. Enzogenol^®^ demonstrated the ability to inhibit all three enzymes: α-amylase enzyme activity (IC_50_ 3.98 ± 0.11 mg/mL), α-glucosidase enzyme activity (IC_50_ 13.02 ± 0.28 μg/mL), and DPP-4 enzyme activity (IC_50_ 2.51 ± 0.04 mg/mL). The present findings indicate the potential for Enzogenol^®^ to improve postprandial glycaemia by delaying carbohydrate digestion via the inhibition of digestive enzymes (α-amylase and α-glucosidase), and enhancing the incretin effect via inhibiting the dipeptidyl-peptidase-4 enzyme. The inhibitory actions of Enzogenol^®^ on enzymes should therefore be further validated in humans for its potential use in type 2 diabetes mellitus prevention and management.

## 1. Introduction

It is estimated that there are currently 537 million (ages 20–79 years) (10.5%) people living with diabetes worldwide, and this number is expected to increase to 783 million (12.2%) by 2045 [1]. Equally alarming, currently 541 million adults (20–79 years) (10.6%) have been estimated to have impaired glucose tolerance (IGT) [1], and this is projected to increase to 730 million (11.4%) by 2045 [1]. Individuals with prediabetes are at high risk for developing diabetes [2]. Alongside genetics, environmental and socioeconomic factors, poor diet choices and sedentary lifestyle contributing to obesity play a major role in the development of type 2 diabetes mellitus (T2D), years before symptoms become visible [1,3,4]. Preventing or retarding the development of T2D by intervening early at the healthy or prediabetes stage may therefore help to delay or minimise the development of diabetic complications and potentially costly long-term drug therapy [5,6].

There is increasing evidence that plant-derived, antioxidant-rich extracts are able to improve glycaemia and may therefore aid in diabetes management and prevention [7,8,9]. In addition, a clear benefit of using antioxidant-rich plants or plant extracts to improve glycaemic control is that there is minimal to no adverse effects as observed with anti-diabetic drug therapy [10]. A recent meta-analysis also demonstrated that using natural health products extracted from plants as an adjunct to anti-diabetic pharmacological therapies further improved measures of blood glucose, particularly in the T2D cohort [11]. Therefore, plant extracts could play a role in diabetes management alongside standard pharmacological therapies.

We have previously demonstrated that Enzogenol^®^ exhibits the ability to significantly improve postprandial glucose in healthy participants, especially those exhibiting monophasic glucose curve shapes indicative of poorer glycaemic control [12]. Additionally, the percentage increment of postprandial glucose (%PG) and 2 h postprandial glucose (2hPG) were significantly reduced with Enzogenol^®^ in healthy participants regardless of their glucose curve shapes. For example, in contrast to control (placebo), 50 mg of Enzogenol^®^ significantly reduced %PG (*p* = 0.003) and 2hPG (*p* = 0.041), and at 400 mg an even higher reductions in %PG (*p* < 0.001) and 2hPG (*p* = 0.012) were observed. However, the specific glucose-lowering mechanisms of Enzogenol^®^ have yet to be fully elucidated.

Inhibiting glucose-regulating enzymes such as α-amylase, α-glucosidase [13,14,15] and dipeptidyl peptidase-4 (DPP-4) [16,17] have been proposed as possible underlying mechanisms by which plant extracts, in particular Enzogenol^®^, exert their hypoglycaemic effects in humans. Notably, inhibiting α-amylase and α-glucosidase responsible for breaking down carbohydrates may prevent or slow carbohydrate digestion during a meal [13,14,15]. Inhibiting DPP-4 enzyme that deactivates incretins that promote glucose-dependent insulin secretion such as the gastric inhibitory polypeptide (GIP) and glucagon-like peptide-1 (GLP-1) may help to preserve their incretin effect in regulating postprandial glycaemia [16,17,18]. Inhibition of these enzymes may therefore potentially lead to improved glycaemic control in humans.

Several in vitro and in vivo studies have investigated the inhibitory effects of other species of pine bark extracts, for example the French Maritime (*Pinus pinaster*, Pcynogenol^®^) and Korean pine bark (*Pinus densiflora*) on α-amylase and α-glucosidase enzymes, although comparative results are inconclusive due to differences in test products and methodologies [19,20,21]. To date, no α-amylase and α-glucosidase inhibition tests have been conducted on Enzogenol^®^ (*Pinus radiata*) that is obtained from New Zealand. Similarly, no DPP-4 inhibition test to date has been conducted on Enzogenol^®^.

Therefore, the aim of the current study was to investigate the potential inhibitory action of Enzogenol^®^ on three key digestive enzymes: α-amylase, α-glucosidase and DPP-4 to determine the underlying mechanistic action of Enzogenol^®^ on postprandial glycaemia.

## 2. Materials and Methods

### 2.1. Materials

Enzogenol^®^ (*Pinus radiata*) was kindly provided by ENZO Nutraceuticals Limited. All chemicals used were of analytical grade. Ultrapure water was prepared from Millipore water purification. Stock phosphate saline buffer (PBS, pH 6.8–6.9) was purchased from Thermo Fisher Scientific. Acarbose, α-amylase from porcine pancreas, α-glucosidase from yeast *Saccharomyces cerevisiae*, 3,5-dinitrosalicylic acid (DNS) reagent, p-nitro-phenyl- α-D-glucopyranoside (p-NPG), sodium carbonate solution (Na_2_CO_3_), and soluble starch (potato), were purchased from Sigma-Aldrich. The DPP-4 inhibitor screening assay kit was purchased from Abcam (Ab133081, Cambridge, UK). The kit consisted of human recombinant DPP-4 enzyme, dipeptidyl-peptidase (DPP) substrate (H-Gly-Pro-AMC), assay buffer (20 mM Tris-HCl, pH 8.0, containing 100 mM NaCl and 1 mM EDTA), and sitagliptin as a positive control inhibitor.

### 2.2. Sample Preparation 

Enzogenol^®^ was prepared using a water-based extraction from the New Zealand *Pinus radiata* trees [22,23]. The dry powder was standardised to contain greater than 80% proanthocyanidins, 1–2% taxifolin, other smaller quantities of flavonoids and phenolic acids [23]. Powdered extract of Enzogenol^®^ was solubilised in dimethyl sulfoxide (DMSO) at 20 mg/mL and diluted with pure Milli-Q water into various concentrations.

### 2.3. Percentage Enzymatic Inhibition and IC_50_ Determination of Enzogenol^®^

Samples of Enzogenol^®^ was sent to Callaghan Innovation, New Zealand for analysis. Alpha-amylase, α-glucosidase and DPP-4 enzyme inhibition assays were conducted on Enzogenol^®^ based on previous studies but with some modifications [24,25]. Different concentrations of the samples and positive controls were tested based on the range possible for the detection of enzymatic inhibitory action. The positive controls acarbose and sitagliptin were run in parallel to ensure assays were working correctly. All samples were run in triplicate. The samples were performed in 96-well plate format on a microreader (SpectraMax 4M, Molecular Devices, LLC., San Jose, CA, USA). The IC_50_ values of Enzogenol^®^ on α-amylase, α-glucosidase, and DPP-4 enzyme inhibition were also determined. The IC_50_ was defined as the concentration required for an inhibitor to reduce 50% of the enzyme activity. The IC_50_ value was obtained from a dose-dependent activity versus concentration plot, where data points of Enzogenol^®^ in five different concentrations were fitted into a non-linear sigmoid plot, accounting for non-linear concentration dependent on enzyme-inhibitor interaction at low and high concentrations.

#### 2.3.1. α-Amylase Activity Inhibition Assay

Enzogenol^®^ was prepared at 0.5, 1, 2.5, 5 and 10 mg/mL. Samples were incubated with α-amylase at 30 °C for 15 min before the addition of 1% starch solution. The hydrolysis of starch by α-amylase in the absence and presence of the sample was kept at 30 °C for 30 min and stopped by adding 1% DNS solution. The mixture was heated at 100 °C for 10 min and then diluted 4-fold with water before readings were measured at 540 nm. The inhibitory effect of α-amylase (%) was calculated using the following formula:

% Inhibition of α-amylase = (1 − As/Ac) × 100,

where As is the absorbance in the presence of sample and Ac is the absorbance of control.

#### 2.3.2. α-Glucosidase Activity Inhibition Assay

Enzogenol^®^ was prepared at 5, 10, 20, 40, and 50 μg/mL. Samples were incubated with α-glucosidase at 37 °C for 15 min. The hydrolytic kinetics by α-glucosidase in the absence and presence of the sample were started by adding p-NPG substrate at 37 °C and monitored at 405 nm for 20 min. The reaction was stopped by adding 0.5 M Na_2_CO_3_ solution and readings were measured at 405 nm. The inhibitory effect of α-glucosidase (%) was calculated using the following formula:

% Inhibition of α-glucosidase = (1 − As/Ac) × 100,

where As is the absorbance in the presence of sample and Ac is the absorbance of control.

#### 2.3.3. Dipeptidyl Peptidase-4 (DPP-4) Activity Inhibition Assay

Enzogenol^®^ was measured at 0.25, 0.5, 1, 2.5 and 5 mg/mL. The reaction was initiated by adding DPP substrate and incubating at 37 °C for 30 min. Readings were measured at Ex 355 nm and Em 460 nm. The inhibitory effect of DPP-4 enzyme was calculated using the following formula:

% Inhibition of DPP-4 enzyme = (Initial activity − Inhibitor/Initial activity) × 100.

### 2.4. Data Analysis

The data were presented as mean ± SEM, with a minimum of *n* = 3 samples of the same batch tested.

## 3. Results

### Percentage Enzymatic Inhibition and IC_50_ of Enzogenol^®^ on Enzymes

Table 1 shows the percentage inhibition activity of Enzogenol^®^ on α-amylase, α-glucosidase and DPP-4 enzymes. The IC_50_ values of Enzogenol^®^ for α-amylase, α-glucosidase and DPP-4 enzymes were 3.98 ± 0.11 mg/mL, 13.02 ± 0.28 μg/mL and 2.51 ± 0.04 mg/mL, respectively.

## 4. Discussion

The present study demonstrated that Enzogenol^®^ exhibited inhibitory effects against α-amylase, α-glucosidase, and DPP-4 enzyme. Enzogenol^®^ had the greatest inhibition on α-glucosidase enzyme followed by DPP-4 enzyme, then α-amylase enzyme. These mechanisms may have important clinical implications for individuals with impaired glycaemic control, by preventing or slowing carbohydrate digestion [13,14,15] and preserving biologically active incretins to stimulate glucose-dependent insulin secretion in the body thereby improving glycaemic control post-meal [17,18].

The hypoglycaemic effect of the New Zealand pine bark may be a result of its unique structural properties such as hydrogen moieties (e.g., OH) and double bonds that determine its effective interaction with digestive enzymes, transmembrane glycoproteins such as DPP-4, apically located transporters and receptors involved in glucose metabolism during a meal [26]. There are two possible mechanisms of enzymatic inhibition that may help explain the underlying inhibitory actions of the New Zealand pine bark. The New Zealand pine bark contains a high concentration of oligomeric proanthocyanidins in varying degrees of polymerisation [23]. Small oligomers of proanthocyanidin may be responsible for the interaction with enzyme proteins in specific active binding cavities during digestion [27]. In contrast, due to their lower bioavailability, larger oligomers and polymers of proanthocyanidin may be accumulated in the small intestine to exert inhibition on enzymes by interacting with enzyme surfaces that result in protein aggregation and precipitation [27,28,29]. A study on a similar species of pine bark (Pycnogenol^®^) concluded that larger oligomers were stronger enzyme inhibitors compared to monomers [19].

Plant extracts have been shown as better inhibitors of α-glucosidase than α-amylase [30,31,32]. Enzogenol^®^ exhibited considerable inhibition against both α-amylase (IC_50_ of 3.98 mg/mL) and α-glucosidase (IC_50_ of 13.02 μg/mL), with a stronger inhibition on α-glucosidase. Similarly, a Korean pine bark extract also showed a higher inhibitory potency against α-glucosidase (IC_50_ of 0.025 μg/mL) compared to α-amylase (IC_50_ of 1.69 μg/mL), although the exact phenolic composition contributing to its inhibitory action was not known [20]. An in vitro study on the French Maritime pine bark also showed strong inhibition of α-glucosidase (IC_50_ of 5.34 μg/mL) [19].

It is noteworthy that differences in test products, methods of extraction and purification, and assay conditions such as pH, reaction time and temperature, substrate concentration and source as well as enzyme concentration and source used in the different studies might have likely contributed to the different IC_50_ values obtained [15,33]. For accurate comparison, the samples should be examined under similar conditions using the same methodology.

Acarbose as an anti-diabetic drug is a strong amylase inhibitor known for causing gastrointestinal adverse effects due to the accumulation of undigested carbohydrates becoming readily available for bacterial fermentation [32,34]. There is therefore potential for Enzogenol^®^, which is a good α-glucosidase and α-amylase inhibitor, to work synergistically with acarbose to inhibit carbohydrate digestion and improve glycaemic control, whilst reducing acarbose dose to alleviate anti-diabetic drug-related adverse effects [30,35,36].

Enzogenol^®^ has also showed inhibitory action against DPP-4 enzyme activity (IC_50_ of 2.51 mg/mL). Currently there is a paucity of data on similar plant extracts, making comparison difficult. The ability of Enzogenol^®^ to inhibit DPP-4 suggests an enhanced incretin effect via a likely increase in active GLP-1 levels. This may help explain the significant improvement in postprandial glucose in healthy participants observed in our previous study [12].

The merits of the present study included understanding the inhibitory action of Enzogenol^®^ on enzymes involved in regulating postprandial glycaemic control. The study outcomes, in particular on DPP-4 enzymatic inhibition, add to the clinical evidence in the observed improvements in postprandial glycaemia in humans [12]. Enzogenol^®^ could therefore potentially be a useful adjunct for those at higher risk for T2D to take alone in conjunction with lifestyle advice. Equally Enzogenol^®^ could also potentially work additively or synergistically when taken together with anti-diabetic drugs such as metformin, often prescribed as a first-line therapy for patients with prediabetes or T2D.

Nevertheless, there are limitations to the study. The present study utilised yeast α-glucosidase that is not precisely biologically similar to mammalian α-glucosidase [13,37]. However due to its availability commercially in a pure form, α-glucosidase has been used as a model for nutraceutical to screen for potential inhibitors [38]. Furthermore, many food/plant extracts have been tested for potential inhibitory action against α-glucosidase deriving from yeast, making this present study suitable to be compared between similar studies [39,40]. This methodology also employed p-NPG substrate that did not allow the differentiation of inhibition against sugar types such as maltose or sucrose in contrast with mammalian α-glucosidase. Future studies could utilise mammalian α-glucosidase obtained from rat intestine that are more similar to human α-glucosidase [30,35].

The in vitro outcomes may also not translate into equivalent outcomes in humans, given the knowledge that plant polyphenols undergo extensive metabolism that may alter their enzymatic inhibitory effects [41,42]. The enzyme inhibitory efficacy of Enzogenol^®^ may also be modified by several dietary factors such as the presence of macronutrients during a meal [43,44]. Therefore, the present findings of Enzogenol^®^ and its inhibitory actions on enzymes should be further validated in humans. The inhibition of Enzogenol^®^ on α-amylase and α-glucosidase could be verified in hydrogen tests [45] and 13C breath tests [46,47,48] in humans after a mixed meal to quantify degree of carbohydrate malabsorption. Similarly, the inhibition of DPP-4 enzyme can be ascertained by measuring concentrations of active circulating incretins (e.g., GLP-1) in plasma samples collected from human participants. 

## 5. Conclusions

Enzogenol^®^ inhibits α-amylase, α-glucosidase and DPP-4 enzymes. The enzymatic inhibitory effects exhibited by Enzogenol^®^ may be one of the mechanisms leading to better glycaemic responses observed in humans. More human studies are warranted to validate the underlying hypoglycaemic mechanistic actions of Enzogenol^®^ and its potential for use in T2D prevention and management.

## Figures and Tables

**Table 1:** Percentage inhibition activity of Enzogenol^®^ on α-amylase, α-glucosidase and DPP-4 enzymes.

α-amylase Enzyme Inhibition					
**Sample (mg/mL)**	**0.5**	**1.0**	**2.5**	**5.0**	**10.0**
**% Inhibition**	5.3 ± 2.7	6.6 ± 0.7	33.8 ± 3.4	58.9 ± 1.6	66.5 ± 5.4
**α-glucosidase enzyme inhibition**					
**Sample (μg/mL)**	**5.0**	**10.0**	**20.0**	**40.0**	**50.0**
**% Inhibition**	13.3 ± 2.6	39.5 ± 4.1	79.6 ± 0.8	98.8 ± 0.03	99.2 ± 0.1
**DPP-4 enzyme inhibition**					
**Sample (mg/mL)**	**0.25**	**0.50**	**1.00**	**2.50**	**5.00**
**% Inhibition**	6.7 ± 3.1	14.0 ± 4.3	21.8 ± 1.7	50.6 ± 0.7	70.5 ± 1.1

## Data Availability

Not applicable.
